# Prevalence and Risk Factors of Depression in Patients With Endemic Osteoarthritis Kashin–Beck Disease

**DOI:** 10.1155/da/8722395

**Published:** 2025-09-09

**Authors:** Ye Liu, Yan Wen, Zhengjun Yang, Ruixue Zhou, Jingni Hui, Cheng Li, Gangyao Xu, Chan Liu, Huan Liu, Bolun Cheng, Yumeng Jia, Xianni Guo, Feng Zhang

**Affiliations:** ^1^Key Laboratory of Trace Elements and Endemic Diseases of National Health and Family Planning Commission, School of Public Health, Health Science Center, Xi'an Jiaotong University, Xi'an, China; ^2^Institute of Endemic Disease Prevention and Control, Shaanxi Provincial Centre for Disease Control and Prevention, Xi'an, Shaanxi, China

**Keywords:** depression, Kashin–Beck disease (KBD), Patient Health Questionnaire-9 (PHQ-9), prevalence

## Abstract

**Background:** Kashin–Beck disease (KBD) is an endemic osteoarthropathy, which occurs in children aged 3–12, with similarity to osteoarthritis (OA). Previous studies have shown significant depression symptoms in OA patients, yet no comparable research has been conducted in KBD patients.

**Methods:** We conducted a field investigation in KBD areas in Northwest China. Questionnaires were designed and used to assess demographic characteristics, clinical characteristics, and medical comorbidities. Patient Health Questionnaire-9 (PHQ-9) was included for the prevalence of depression. Finally, 440 subjects were clinically diagnosed and recruited. Depression was diagnosed when PHQ-9 ≥ 5 and classified into mild (5–9), moderate (10–14), and severe (≥ 15) groups. Logistic regression was also used to identify potential associated factors among KBD patients.

**Results:** Depression was present in 53.2% of patients in our KBD samples. Among them, 27.5% had mild depression, 18.4% had moderate depression, and 7.3% had severe depression. Being male (odds ratio [OR]: 0.296, 95% confidence interval [CI]: 0.180–0.486, *p* < 0.001) was an independent protective factor for depression, while the presence of comorbid chronic diseases (OR: 4.701, 95% CI: 2.292–9.640, *p* < 0.001), and a higher visual analog scale (VAS) pain level (OR: 5.275, 95% CI: 1.326–20.978, *p*=0.018) were independent risk factors for depression in KBD patients.

**Conclusion:** This study is the first to investigate the prevalence of depression and associated factors among Chinese KBD patients, suggesting the significance of an early intervention for their mental issues.

## 1. Introduction

**Figure 1:**
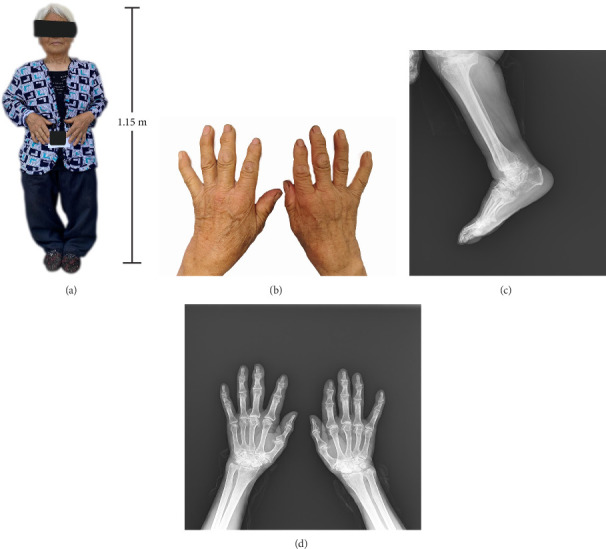
Patient with III-degree KBD and radiographic features in the hand and ankle region. (A) Full-body photograph of a 78-year-old, 1.15-m-tall patient with III-degree KBD. (B) and (D) Hands with brachydactyly and its radiograph. (C) Right ankle radiograph.

Kashin–Beck disease (KBD) is a severe degenerative chronic joint disease, leading to irreversible pathological and clinical manifestations. Its typical symptoms include symmetrical enlargement of the phalanges, brachydactyly, joint deformities, and even dwarfism [1, 2] (Figure 1). Patients with advanced KBD often partially or completely lose their ability to work and perform self-care, severely impacting their life quality and increasing the economic burden on their families [3]. KBD is primarily endemic in a diagonal region from northeast to southwest China, with additional endemic areas in Siberia and North Korea [4]. According to the 2020 national survey of China, there are over 170,000 KBD patients, with most patients being over 50 years old, and more than 20 million people are under the threaten of KBD [5].

Joint diseases involve complex systemic pathological processes, with recent studies highlighting a bidirectional communication between the brain and joints [6], referred to as the joint–brain axis [7]. This axis refers to the mutual influence between joint diseases and the central nervous system (CNS), facilitated through inflammatory mediators, neural signaling, and immune responses [7]. In osteoarthritis (OA) patients, chronic pain and inflammation significantly affect brain function and structure [8], extending to neuroinflammation linked to symptoms like depression, anxiety, and cognitive impairment [9]. Additionally, altered neurotransmitter signaling, such as glutamate and serotonin, impacts pain perception and cognitive function [7]. OA patients who experienced chronic pain showed reduced gray matter in key brain regions [10] associated with pain perception [11] measured by functional magnetic resonance imaging (fMRI).

Although advanced KBD and OA have similar symptoms, they differ in several key aspects, including onset age, clinical symptoms, and X-ray performance [12]. KBD typically begins early at 3–12 years old, causing joint deformities such as wrist radial deviation, brachydactylia, and shortened heels [12], while OA usually develops later in life, commonly after the age of 50, and is characterized by joint pain, swelling, and osteophyte formation [13]. These differences are essential for differential diagnosis and highlight the distinct developmental trajectory of KBD. Compared to OA patients, KBD's early onset during childhood [14] and chronic joint inflammation [15] may have a more profound impact on the brain due to the joint–brain axis. Impaired brain maturation [14] and inflammation [16] during childhood may contribute to more severe cognitive impairments and a higher prevalence of mental disorders in KBD patients.

Therefore, this study aims to systematically evaluate depression in KBD patients. Additionally, we explore the association between different depression groups and disease activity, functional impairment, and demographic characteristics. Through this research, we aim to enhance the understanding of mental health issues in KBD patients and provide a scientific basis for developing effective intervention strategies.

## 2. Methods

### 2.1. Study Design and Sample Collection

This study was conducted in Lantian County, a high-prevalence endemic area for KBD in China. A total of 440 KBD patients were included in the final analysis. All patients were Shaanxi Han Chinese with a similar geographic background. KBD patients were diagnosed according to the national diagnostic criteria of KBD in China (WS/T 207-2010), which are based on residence in endemic areas, clinical manifestations, and characteristic X-ray findings (such as defects and sclerosis at the phalangeal bone ends, compression changes in the calcaneus and talus, and enlarged or deformed limb joints). Exclusion criteria included having any other bone and joint disease (e.g., rheumatoid arthritis [RA], gout, OA, osteochondrodysplasia), severe alterations in joint lines of force (e.g., X-legs, O-legs), the presence of joint free-form bodies, severe joint effusions, a diagnosis of malignancy, or a history of psychiatric disorders and the use of psychotropic medications. During the field investigation, individuals who were willing to participate were evaluated by experienced KBD specialists, and only those who were clinically diagnosed with KBD and met all eligibility criteria were ultimately invited to participate in the study. Therefore, the final sample included 440 patients with confirmed KBD. All participants were instructed to discontinue any medications related to KBD for at least 14 days to avoid the potential confounding effect of pain relief medications. This study has been approved by the human ethics committee of Xi'an Jiaotong University (No. 2022-1375). All subjects signed a written informed consent form.

### 2.2. Data Collection

All investigators received comprehensive training designed to ensure a thorough understanding of the survey's purpose and significance, the structure and definitions of the questionnaire, and relevant knowledge. This training aimed to standardize the interpretation and completion of the indicators and clarify the survey workflow and necessary precautions. Data collection was conducted in a quiet environment, free from external interference, to maintain the integrity of the responses. Trained investigators collected the general clinic data during face-to-face interviews with patients, including age, sex, educational background, comorbidities types, disease characteristics, such as KBD degree, joint dysfunction score, and the visual analog scale (VAS) pain score. Trained nurse measured height and weight and calculated the body mass index (BMI) of KBD patients. Detailed information was shown in the Supporting Information.

### 2.3. Assessment of Depression

We used the Patient Health Questionnaire-9 (PHQ-9) to assess the depression of KBD patients, which is a screening tool that measures the presence and severity of depressive symptoms. The PHQ-9 is a self-report questionnaire that consists of nine items based on the Diagnostic and Statistical Manual of Mental Disorders-IV (DSM-IV) criteria for assessing symptoms of depression [17]. The questionnaire assesses whether the listed symptom has occurred or bothered the subject during the last 2 weeks. Each item of the PHQ-9 scores from 0 to 3, with a summed score of 0–27 for the nine items. By calculating a summary score, the severity of a depressive episode can be assessed. Scores of 0–4 indicate no depression, 5–9 indicate mild depression, 10–14 indicate moderate depression, and ≥ 15 indicate moderately severe depression [18]. Based on the generally recommended criterion, a cutoff point of ≥ 5 has a sensitivity of 81.5% and a specificity of 80.6% for mild depression [19]. The Chinese version of the PHQ-9 has demonstrated good psychometric properties, with both sensitivity and specificity of 0.86, supporting its validity and efficiency as a screening tool for depression in the general Chinese population [18].

### 2.4. Statistical Analysis

The normality of the continuous variables was tested using the Kolmogorov–Smirnov test. For count data expressed as frequency and percentage (*n* [%]), the differences between groups were analyzed by the chi-square test. Continuous variables that conformed to a normal distribution were expressed as mean ± standard deviation (mean ± SD), and differences between groups were analyzed by analysis of variance (ANOVA). Otherwise, the median and interquartile spacing (M [Q1, Q3]) were used, and nonparametric tests were employed. The rank sum test was employed for ordered categorical data, whereas the chi-square test was utilized for unordered categorical data.

Those variables with significant differences emerging in the comparative analyses were included in a series of logistic regression models, using the forward conditional method, to determine which variables could predict the presence of depression in patients with KBD. *β* value, standard error (SE) of *β*, odds ratio (OR), and 95% confidence interval (CI) were calculated for each model. A *p*-value less than 0.05 was considered statistically significant. All analyses were conducted in IBM SPSS Statistics 25.0.

## 3. Results

### 3.1. Sample Description

A total of 440 individuals with clinically confirmed KBD were included in the analysis. Baseline characteristics are shown in Table 1. The median age was 69 years (IQR: 62–73), ranging from 40 to 86 years. The median height was 151.2 cm (IQR: 145.0–158.0), and the median weight was 56.0 kg (IQR: 49.3–62.0), corresponding to a median BMI of 24.2 kg/m^2^ (IQR: 21.5–27.1). Regarding sex distribution, 213 (48.4%) were male and 227 (51.6%) were female.

The median PHQ-9 score was 5 (IQR: 2–10), with 53.2% of patients meeting the threshold for depression (PHQ-9 ≥ 5). Among them, 27.5% had mild depression, 18.4% moderate depression, and 7.3% severe depression.

### 3.2. Univariate Analysis Between KBD Patients With or Without Depression

Demographic and clinical characteristics according to the presence of depression are shown in Table 2. KBD patients with or without depression had differences in sex (*p* < 0.001), educational background (*p*=0.009), number of comorbidities (*p* < 0.001), VAS pain level (*p* < 0.001), and joint dysfunction score (*p* < 0.001). Male patients, those who have a higher educational background and lower comorbidities, lower VAS pain level, and lower joint dysfunction score, had a lower probability of depression. However, age, BMI, and KBD degree showed no significant differences between groups (*p* > 0.05).

### 3.3. Predictors of Depression in KBD Patients

A multivariate logistic regression analysis was conducted. The presence of depression was used as the dependent variable, while the characteristics that differed in the univariate analysis were used as the independent variables.

The regression results were shown in Table 3, indicating that being male (OR: 0.296, 95% CI: 0.180–0.486, *p* < 0.001) was an independent protective factor for depression in KBD patients, while the presence of comorbid chronic diseases (OR: 4.701, 95% CI: 2.292–9.640, *p* < 0.001), and a higher VAS pain level (OR: 5.275, 95% CI: 1.326–20.978, *p*=0.018) were independent risk factors for depression in KBD patients. Although the educational background and joint dysfunction score showed no significance, it was observed that the presence of depression decreases under higher levels of education and lower joint dysfunction scores. Among these, senior high school (OR = 0.828) was identified as a protective factor, while a higher joint dysfunction score (OR = 1.612) was identified as a risk factor.

## 4. Discussion

The purpose of this study is to quantify the prevalence of depression among KBD patients and analyze the demographic characteristics and disease indicators influencing these conditions. In our sample, 53.2% exhibited depression based on the PHQ-9 cutoff points. As hypothesized, our results showed a high prevalence of depression among KBD patients, particularly among females, those with a higher number of comorbid chronic diseases, and those experiencing more severe pain. These findings suggest the problem of depression popularity among KBD, which should be taken into consideration when designing and proposing the intervention strategy for KBD.

The clinical manifestations of KBD primarily include pain, restricted mobility, and joint deformities, making it a special type of OA [15]. Like the clinical presentations of OA, it is often difficult to distinguish KBD from OA [20]. The prevalence of depression in OA patients varies across different studies, depending on the population assessed, the screening tools used, and the definitions of depression [21]. A systematic review found that the overall prevalence of depression in OA patients is 19.9% (95% CI: 15.9%–24.5%). Additionally, the prevalence of depression in adult knee OA (KOA) patients and those with mixed lower limb OA is 18.5% (95% CI: 13.8%–23.7%) and 23.0% (95% CI: 16.4%–30.2%), respectively [22]. The National Health Interview Survey (2015–2017) revealed that the age-standardized prevalence of depressive symptoms in adults with arthritis was 12.1%, in comparison to 4.7% in adults without arthritis [23]. A study conducted in Changsha, China, reported a mean PHQ-9 score of 4.72 ± 4.39 among elderly patients with KOA, indicating the common presence of depressive symptoms in this population [24]. Another study conducted in Australia among 397 patients with OA reported that the overall prevalence of depression, defined by a PHQ-9 score ≥ 5, was 25.4% [19]. A cross-sectional study conducted in Nigeria among 250 patients with KOA reported that 42% of the participants had depression, defined by a PHQ-9 score ≥ 5 [25]. In the German primary care population, nearly 20% of OA patients were found to have moderate to severe depression, with an average PHQ-9 score exceeding 15, indicating a relatively high level of depressive symptoms [26].

Our study found that compared to OA patients, KBD patients experience more severe depressive symptoms. This difference may be attributed to several factors. First, while OA typically affects older adults, KBD onset commonly occurs between ages 3 and 12 [27], a critical period for brain development. The prolonged disease course of KBD means that patients are exposed to chronic joint damage, pain, and inflammation throughout developmental stages, possibly leading to long-term alterations in brain function. Studies have reported that the quality of life in KBD patients is markedly lower than in OA patients and healthy individuals [28], which may further exacerbate emotional distress.

Second, inflammation-related biological mechanisms may further explain the elevated risk of depression among KBD patients. In recent years, joint–brain axis suggests that chronic joint disorders may influence not only local joint structures but also the CNS through multiple interconnected pathways [7, 29]. These include sustained elevation of peripheral proinflammatory cytokines (e.g., IL-1β, IL-6, and TNF-α), altered neural signaling [9], and prolonged immune activation [6, 7, 30]. Such cytokines can reach the brain via several routes, including afferent vagal pathways, cerebral vascular endothelium, and circumventricular organs, ultimately leading to neuroinflammation [6, 7, 9]. For example, research has demonstrated that chronic pain in OA patients is associated with gray matter volume reduction in key brain regions involved in emotion and cognitive processing, including bilateral amygdaloid and cerebellum posterior lobe [31]. In RA, neuroinflammation is associated with impaired neuronal function. Several mechanisms have been proposed, including alterations in neurotransmitter signaling; dynamic modulation of dendritic spines and neuronal networks; and impaired adult hippocampal neurogenesis [7], which correlate with symptoms of depression, anxiety, and cognitive impairment [32]. Moreover, vagus nerve stimulation was found to be able to attenuate RA symptoms by modulating inflammatory responses, suggesting a feedback role of the CNS in regulating peripheral inflammation [7, 33]. Although traditionally considered noninflammatory, KBD has been shown to involve low-grade chronic inflammation, which stimulates proinflammatory cytokines, such as IL-1β, IL-6, and TNF-α [15], suggesting that similar pathways may be involved.

Moreover, the onset of KBD during early childhood coincides with critical periods of neurodevelopment, during which the brain is particularly vulnerable to external insults. Disruptions caused by inflammatory cytokines, chronic pain, or reduced physical and social engagement may impair the maturation of brain regions responsible for emotion and cognition, such as the prefrontal cortex and limbic system [14, 34]. In addition, many KBD patients—particularly those living in rural endemic areas—face persistent limitations in access to education, medical care, and psychosocial support. This combination of early-onset physical disability, social stigma, and environmental deprivation aligns closely with the framework of early-life adversity, a well-established risk factor for depression in later life [34]. These cumulative psychosocial stressors, when occurring alongside long-term biological disruption, may synergistically contribute to the elevated prevalence and severity of depression observed in the KBD population. Further research incorporating neuroimaging, developmental assessments, and inflammatory biomarkers is warranted to elucidate the interplay between the joint–brain axis and early-life adversity in shaping mental health outcomes in this unique population.

Univariate logistic analysis found that KBD joint dysfunction scores, VAS scores, comorbidities, and sex were associated with depression. Some key factors affecting the health-related quality of life in KBD patients have been identified, such as age, education level, severity of KBD, and economic status [4]. Similarly, studies on OA identified risk factors for depression, typically divided into those related to disease severity and general factors [21]. Regarding disease severity, a longitudinal study by Veronese et al. [35] found that individuals with multiple OA sites and those with lower limb OA were more likely to experience depression. Sugai et al. [36] observed that severe knee pain (night pain) and dysfunction (difficulty putting on socks, getting in and out of a car, taking off socks) significantly increased the risk of depression. Zheng et al. [19] found that higher baseline Western Ontario and McMaster Universities Arthritis Index (WOMAC) pain and dysfunction scores, along with two or more pain sites, were significantly associated with a higher incidence of depression. As for general factors, longitudinal studies have identified females, low income, and smoking as predictors of rapidly worsening depression in KOA patients [37]. Other cross-sectional studies have similarly found that OA patients reporting depression are more likely to be female, have a higher BMI, have more comorbidities, and have a lower age [26, 38].

This study has several strengths. First, it was conducted in a real-world clinical setting, enhancing the generalizability of the findings. Second, the sample size is one of the largest observational studies of depressive symptoms in KBD patients. However, there are limitations to consider. The cross-sectional design necessitates caution in making causal inferences. Selection bias cannot be entirely ruled out due to the study design. The outcome measures are self–reported and should not be interpreted as truly objective assessments of depressive symptoms. It is crucial to acknowledge the potential for unmeasured confounding factors, such as medication use, smoking, and alcohol consumption, that were not collected in the study.

In summary, our findings indicate a high prevalence of depressive symptoms among KBD patients. By drawing on insights from OA studies, we can better understand and address the psychological issues in KBD patients. The current treatment goals for KBD in China are to improve joint dysfunction, alleviate pain, and enhance quality of life. These findings underscore the necessity for mental health assessments in KBD patients, as well as the development of appropriate interventions to improve their overall quality of life.

## Figures and Tables

**Table 1:** Baseline characteristics.

Characteristics	*M* (Q1, Q3)	Range
Age (year)	69 (62, 73)	40–86
Height (cm)	151.2 (145.0, 158.0)	115.5–178.0
Weight (kg)	56 (49.3, 62.0)	29.4–95.0
BMI (kg/m^2^)	24.2 (21.5, 27.1)	15.4–44.1
Joint dysfunction score	7 (5, 9)	0–10
VAS pain score	6 (3, 7)	0–10
PHQ-9 score	5 (2, 10)	0–27

	***N* (%)**	

Sex		
Male	213 (48.4%)	—
Female	227 (51.6%)	—
Educational background		
Illiteracy	125 (28.4%)	—
Elementary school	131 (29.8%)	—
Junior high school	149 (33.9%)	—
Senior high school	33 (7.5%)	—
University/college	0	—
Number of comorbidities		
0	103 (23.4%)	—
1	148 (33.6%)	—
2	106 (24.1%)	—
≥3	83 (18.9%)	—
KBD degree		
I	326 (74.1%)	—
II	97 (22.0%)	—
III	17 (3.9%)	—
VAS pain level		
No pain	17 (3.9%)	—
Mild pain	94 (21.4%)	—
Moderate pain	165 (37.5%)	—
Severe pain	164 (37.3%)	—
Depression groups		
No depression	206 (46.8%)	—
Mild depression	121 (27.5%)	—
Moderate depression	81 (18.4%)	—
Severe depression	32 (7.3%)	—

Abbreviations: BMI, body mass index; KBD, Kashin–Beck disease; PHQ-9, Patient Health Questionnaire-9; VAS, visual analog scale.

**Table 2:** Comparison of demographic and clinical characteristics of KBD patients with or without depression.

Characteristics	*N*	No depression	Depression	*p*
		*N* = 206	*N* = 234	
Sex				
Male	213	133 (62.4%)	80 (37.6%)	<0.001
Female	227	73 (32.2%)	154 (67.8%)	—
Age				
<60	77	34 (44.2%)	43 (55.8%)	0.696
60–70	160	74 (46.3%)	86 (53.8%)	—
70–80	175	82 (46.9%)	93 (53.1%)	—
≥80	28	16 (57.1%)	12 (42.9%)	—
BMI				
<18.5	18	7 (38.9%)	11 (61.1%)	0.301
18.5–23.9	197	100 (50.8%)	97 (49.2%)	—
≥24	225	99 (44.0%)	126 (56.0%)	—
Educational background				
Illiteracy	125	51 (40.8%)	74 (59.2%)	0.009
Elementary school	131	52 (39.7%)	79 (60.3%)	—
Junior high school	149	81 (54.4%)	68 (45.6%)	—
Senior high school	33	21 (63.6%)	12 (36.4%)	—
Number of comorbidities				
0	103	67 (65.0%)	36 (35.0%)	<0.001
1	148	76 (51.4%)	72 (48.6%)	—
2	106	41 (38.7%)	65 (61.3%)	—
≥3	83	22 (26.5%)	61 (73.5%)	—
KBD degree				
I	326	157 (48.2%)	169 (51.8%)	0.594
II	97	41 (42.3%)	56 (57.7%)	—
III	17	8 (47.1%)	9 (52.9%)	—
VAS pain level				
No pain	17	13 (76.5%)	4 (23.5%)	<0.001
Mild pain	94	72 (76.6%)	22 (23.4%)	—
Moderate pain	165	79 (47.9%)	86 (52.1%)	—
Severe pain	164	42 (25.6%)	122 (74.4%)	—
Joint dysfunction score				
0–5	115	79 (68.7%)	36 (31.3%)	<0.001
6–10	325	127 (39.1%)	198 (60.9%)	—

Abbreviations: BMI, body mass index; KBD, Kashin–Beck disease; VAS, visual analog scale.

**Table 3:** Multivariate logistic regression models with the forward conditional method for the prediction of depression in KBD patients.

Characteristics	*β*	SE	Wald	*p*	OR	95% CI
Sex						
Female	0	—	—	—	1.000	—
Male	−1.217	0.253	23.156	<0.001	0.296	0.180–0.486
Educational background						
Illiteracy	0	—	—	—	1.000	—
Elementary school	0.248	0.300	0.684	0.408	1.282	0.712–2.308
Junior high school	0.120	0.318	0.143	0.706	1.128	0.604–2.104
Senior high school	−0.189	0.480	0.156	0.693	0.828	0.323–2.119
Number of comorbidities						
0	0	—	—	—	1.000	—
1	0.763	0.301	6.433	0.011	2.146	1.189–3.871
2	1.219	0.337	13.063	<0.001	3.385	1.747–6.559
≥3	1.548	0.366	17.841	<0.001	4.701	2.292–9.640
VAS pain level						
No pain	0	—	—	—	1.000	—
Mild pain	−0.319	0.696	0.209	0.647	0.727	0.186–2.847
Moderate pain	0.658	0.691	0.907	0.341	1.931	0.498–7.486
Severe pain	1.663	0.704	5.575	0.018	5.275	1.326–20.978
Joint dysfunction score						
0–5	0	—	—	—	1.000	—
6–10	0.478	0.297	2.581	0.108	1.612	0.900–2.887

*Note: β*, the regression coefficient.

Abbreviations: CI, confidence interval; OR, odds ratio; SE, standard error; VAS, visual analog scale.

## Data Availability

The datasets used and/or analyzed during the current study are available from the corresponding author upon reasonable request.

## Nomenclature

KBD: Kashin–Beck disease
PHQ-9: Patient Health Questionnaire-9
VAS: Visual analog scale
CNS: Central nervous system
OA: Osteoarthritis
RA: Rheumatoid arthritis
fMRI: Functional magnetic resonance imaging
BMI: Body mass index
DSM-IV: Diagnostic and Statistical Manual of Mental Disorders-IV
SE: Standard error
OR: Odds ratio
CI: Confidence interval
WOMAC: Western Ontario and McMaster Universities Arthritis Index.

## Supporting Information

Supporting Information The supporting Information provides detailed descriptions of the following components: comorbidities, Joint dysfunction score, visual analog scale, and KBD disease degree.
