# Supplementary material for: Prevalence and Risk Factors of Depression in Patients With Endemic Osteoarthritis Kashin–Beck Disease
Source: Depress Anxiety. 2025 Sep 9;2025:8722395. doi: 10.1155/da/8722395 (PMC12440642; doi:10.1155/da/8722395)
Supplement: Supporting Information — The supporting Information provides detailed descriptions of the following components: comorbidities, Joint dysfunction score, visual analog scale, and KBD disease degree. [file 8722395.f1.docx]

**Measurements of co–morbidities in detail**

Comorbidities were assessed based on participants’ self-reported medical history, confirmed through local health records when available. The conditions included were hypertension, diabetes mellitus, coronary heart disease, cerebral infarction, chronic respiratory diseases, digestive system diseases, and thyroid disorders. Patients with severe systemic diseases such as cancer were not included in this study.

**Measurements of Joint dysfunction score in detail**

The joint dysfunction score was assessed based on the "Criteria for Evaluating the Therapeutic Effect of Kashin–Beck Disease" [WS/T 79–2011]. It includes five indicators: Joint rest pain, Joint motion pain, Morning stiffness, Maximum walking distance, and Lower limb mobility. Each item is rated on a three-level scale: "0", "1", and "2", corresponding to scores of 0, 1, and 2, respectively. The total score is the sum of all five indicators, with higher scores indicating more severe joint dysfunction.

| **Terms** | **Standard** | **Score** |
| --- | --- | --- |
| Joint rest pain | None | 0 |
|  | Pain present but does not affect sleep | 1 |
|  | Pain affects sleep, necessitating the use of painkillers | 2 |
| Joint motion pain | None | 0 |
|  | Pain occurs when going up or down slopes (stairs) or walking for more than 15 minutes. | 1 |
|  | Pain is significant and unbearable when going up or down slopes (stairs) or walking for less than 15 minutes. | 2 |
| Morning stiffness | None | 0 |
|  | Morning joint stiffness lasts less than 15 minutes | 1 |
|  | Morning joint stiffness lasts more than 15 minutes | 2 |
| Maximum walking distance | Can walk more than 1000 meters without limitations | 0 |
|  | Can only walk 500 to 1000 meters | 1 |
|  | Can walk less than 500 meters or only within home/yard | 2 |
| Lower limb mobility | Squats easily | 0 |
|  | Difficulty squatting or knee flexion less than 90° | 1 |
|  | Cannot squat or knee flexion greater than 90° | 2 |
| Joint dysfunction score | | |

We sum the scores of the five items to obtain the joint dysfunction score range from 0-10. A higher score indicates worse joint function. 1-5 considered low joint dysfunction, 6-10 considered high joint dysfunction.

**Measurements of visual analog scale in detail**

The visual analog scale (VAS) pain score which were categorized the VAS scores (range: 0–10) into four levels according to widely used criteria: No pain (0), Mild pain (1–3), Moderate pain (4–6), Severe pain (7–10).

**Measurements of KBD disease degree in detail**

The KBD disease degree information was obtained from the local Center for Disease Prevention and Control. According to the national diagnostic criteria for Kashin–Beck disease [WS/T 207–2010], KBD severity is clinically classified into three grades:

Grade I: Characterized by multiple and symmetrical thickening of finger joints, thickening of other limb joints, limited flexion and extension, joint pain, and mild muscle atrophy.

Grade II: Based on Grade I, symptoms and signs become more severe, with the appearance of deformities such as brachydactyly (shortened fingers or toes).

Grade III: On top of Grade II symptoms, patients show further aggravation, including dwarfism and more pronounced joint and skeletal deformities.
